# Dorsal Striatum Dopamine Levels Fluctuate Across the Sleep–Wake Cycle and Respond to Salient Stimuli in Mice

**DOI:** 10.3389/fnins.2019.00242

**Published:** 2019-03-19

**Authors:** Hui Dong, Juan Wang, Yan-Fei Yang, Yan Shen, Wei-Min Qu, Zhi-Li Huang

**Affiliations:** ^1^Department of Pharmacology, School of Basic Medical Sciences, State Key Laboratory of Medical Neurobiology and MOE Frontiers Center for Brain Science, Institutes of Brain Science, Fudan University, Shanghai, China; ^2^Department of Neurology and National Research Center for Aging and Medicine, Huashan Hospital, Fudan University, Shanghai, China

**Keywords:** dopamine, dorsal striatum, sleep–wake, dLight, modafinil

## Abstract

Dopamine is involved in numerous neurological processes, and its deficiency has been implicated in Parkinson’s disease, whose patients suffer from severe sleep disorders. Destruction of nigrostriatal dopaminergic neurons or dorsal striatum disrupts the sleep–wake cycle. However, whether striatal dopamine levels correlate with vigilance states still remains to be elucidated. Here, we employed an intensity-based genetically encoded dopamine indicator, dLight1.1, to track striatal dopamine levels across the spontaneous sleep–wake cycle and the dopaminergic response to external stimuli. We found that the striatal dLight1.1 signal was at its highest during wakefulness, lower during non-rapid eye movement (non-REM or NREM) sleep, and lowest during REM sleep. Moreover, the striatal dLight1.1 signal increased significantly during NREM sleep-to-wake transitions, while it decreased during wake-to-NREM sleep transitions. Furthermore, different external stimuli, such as sudden door-opening of the home cage or cage-change to a new environment, caused striatal dopamine release, whereas an unexpected auditory tone did not. Finally, despite both modafinil and caffeine being wake-promoting agents that increased wakefulness, modafinil increased striatal dopamine levels while caffeine did not. Taken together, our findings demonstrated that striatal dopamine levels correlated with the spontaneous sleep–wake cycle and responded to specific external stimuli as well as the stimulant modafinil.

## Introduction

Dopamine is involved in numerous behavioral and psychological processes, including motor behavior, attention, motivation, reward, and feeding (Palmiter, 2007; Berke, 2018), all of which operate on the basis of wakefulness (Lazarus et al., 2012, 2013). Dysregulation of the striatum and nigrostriatal dopamine are considered to be responsible for Parkinson’s disease (PD). Patients with PD have been reported to suffer from severe sleep disorders including insomnia, sleep fragmentation, excessive daytime sleepiness (EDS), and rapid eye movement (REM) sleep behavior disorders (Adler and Thorpy, 2005). Lesioning the dorsal striatum decreases and destabilizes wakefulness in rats (Qiu et al., 2010). The dorsal striatum expresses dopamine D_1_ and D_2_ receptors (D_1_Rs, D_2_Rs) at high levels (Weiner et al., 1991; Levey et al., 1993). D_1_R and D_2_R agonists have been shown to dramatically promote wakefulness (Ongini et al., 1985; Monti et al., 1989). Moreover, our previous study showed that genetic deletion of D_2_Rs significantly decreases wakefulness in mice (Qu et al., 2010). These findings suggest that nigrostriatal dopamine is crucial for wakefulness.

The striatum receives dense dopaminergic inputs from the substantia nigra pars compacta (SNc), and partially from the ventral tegmental area (VTA) and dorsal raphe nucleus (DRN) (Stratford and Wirtshafter, 1990; Bjorklund and Dunnett, 2007; Wall et al., 2013; Poulin et al., 2018). Recent evidence reveals that dopaminergic neurons in the SNc, VTA, and DRN are pivotal for the initiation and maintenance of wakefulness (Eban-Rothschild et al., 2016; Cho et al., 2017; Oishi et al., 2017a; Yang et al., 2018). Optogenetic or chemogenetic stimulation of dopaminergic neurons in the SNc, VTA, or DRN induces robust wakefulness (Eban-Rothschild et al., 2016; Cho et al., 2017; Oishi et al., 2017a; Yang et al., 2018). The calcium activity of dopaminergic neurons is demonstrated to be high during wakefulness and correlates with state transitions (Eban-Rothschild et al., 2016; Cho et al., 2017). However, previous studies showed that dopaminergic neurons in the SNc and VTA not only release dopamine but also co-release either glutamate or γ-aminobutyric acid (GABA) (Chuhma et al., 2004; Hnasko et al., 2010; Tritsch et al., 2012; Kim et al., 2015). In addition, activation of dopaminergic fibers in striatal slices rapidly inhibits the action potential firing of striatal medium spiny neurons (MSNs) via the release of the inhibitory transmitter GABA (Tritsch et al., 2012). Early electrophysiological findings suggest that dopaminergic neurons in the VTA and SNc do not change their mean firing rate and pattern across sleep–wake states in rats and cats (Trulson et al., 1981; Miller et al., 1983; Steinfels et al., 1983; Trulson and Preussler, 1984). The lesion of VTA and SNc dopaminergic neurons in cats results in a lack of behavioral arousal but not the alteration of electrocortical waking (Jones et al., 1973). Despite numerous studies devoted to how dopaminergic neurons and dopamine receptors are vital for wakefulness, the field still lacks straightforward and detailed evidence to support that dopamine itself plays a role in the sleep–wake cycle. To address this question, methods with high temporal resolution are needed to monitor the variation of striatal dopamine levels across the sleep–wake cycle.

Classical analytical approaches such as intracerebral microdialysis and electro-chemical voltammetry have been used for the quantitative measurement of extracellular dopamine concentrations, but they provide poor temporal resolution. Using intracerebral microdialysis with a 2-min temporal resolution, a previous study found that dopamine concentrations in the nucleus accumbens (NAc) and prefrontal cortex (PFC) are higher during both the awake state and REM sleep compared to non-REM (NREM) sleep in rats (Lena et al., 2005). Another study using voltammetry at a 5-min resolution showed that the striatal dopamine voltammetric peak is higher in cats while awake than asleep (Trulson, 1985). In addition, extracellular dopamine levels in mouse striatal slices oscillates across the light/dark cycle (Ferris et al., 2014). The above methods have provided useful insights about the release of dopamine transmitter, but poor temporal resolution in freely moving animals still presents a significant limitation. Recently, Patriarchi et al. (2018) engineered a genetically encoded fluorescent dopamine sensor, dLight1.1, which is capable of tracking dopamine transients with high temporal resolution in freely moving animals. The dLight1.1 sensor is developed by replacing the third intracellular loop on D_1_R with a circularly permuted GFP (cpGFP) and permits the tracking of dopamine levels by detecting cpGFP fluorescence without activating D_1_Rs signaling cascades downstream.

In our current study, we employed an optimized variant of this dopamine sensor called dLight1.1, which is suitable for *in vivo* studies. We detected the dLight1.1 fluorescent signals using fiber photometry, while simultaneously collecting polysomnographic recordings in freely behaving mice after environmental or pharmacological manipulations. We found that striatal dopamine levels were at their highest during wakefulness, lower during NREM sleep, and lowest during REM sleep. We also revealed that striatal dopamine levels were correlated with sleep-state transitions. Furthermore, dopamine levels were enhanced in the striatum following the sudden opening of the home-cage door but did not respond to a high-frequency auditory stimulus whether asleep or awake. Moving the mice from their home cage to a new cage also caused striatal dopamine release. Finally, the wake-promoting agent modafinil, but not caffeine, induced the release of striatal dopamine. Taken together, our results provided strong evidence that striatal dopamine levels correlated with wakefulness and could respond to defined stimuli and stimulants.

## Materials and Methods

### Ethics Statement

This study was carried out in accordance with the principles of China Regulations on the Administration of Laboratory Animals, the Decree NO.2 of National Science and Technology Commission of the People‘s Republic of China. The protocol was approved by the Committee on the Ethics of Animal Experiments of Fudan University (permit number: 20140226-024).

### Animals

Male, specific pathogen-free (SPF), inbred C57BL/6 mice (10–14 weeks old weighing 20–25 g) were obtained from the Shanghai Laboratory Animal Center, Chinese Academy of Sciences (SLAC, Shanghai, China). The mice were housed at a constant temperature (22 ± 0.5°C and humidity (55 ± 5%), under an automatically controlled 12/12 h light/dark cycle (lights on at 7:00 a.m., illumination intensity ≈ 100 lux) (Zhang et al., 2017). Food and water were available *ad libitum*. Every effort was made to minimize animal suffering, and the minimum number of animals required to generate reliable scientific data was used.

### Virus Preparation

The adeno-associated virus (AAV) plasmid pAAV-CAG-dLight1.1 was a gift from Lin Tian (Addgene plasmid # 111067) (Patriarchi et al., 2018). A recombinant AAV vector carrying the dLight1.1 element (AAV-CAG-dLight1.1) was serotyped with AAV9 coat proteins and packaged by Taitool Bioscience Company (Shanghai, China). The final viral concentration was 5 × 10^12^ genome copies per mL. Aliquots of virus were stored at -80°C until stereotaxic injection.

### Viral Microinjection and Optical-Fiber Cannula Implantation

Adult mice were anesthetized with pentobarbital (intraperitoneal, 80 mg/kg) and 1% lidocaine hydrochloride (subcutaneous, under the scalp). After shaving the fur on the head and sterilizing the skin with 75% ethanol, the mice were placed on a stereotaxic frame (RWD Life Science, China). The skull surface was cleaned with sterile saline on a sterilized cotton swab. Small craniotomy burr holes were made and 100 nL of the AAV-CAG-dLight1.1 virus was unilaterally microinjected through a fine glass pipette into the dorsal striatum (anteroposterior (AP): 0.80 mm, mediolateral (ML): +1.5 mm, dorsoventral (DV): -2.5 mm), according to the Allen Mouse Brain Atlas (Dong, 2008). The virus injection was administered over a 5-min period using nitrogen-gas pulses of 20 psi delivered through an air compression system (Picospritzer III, Parker Hannifin Corp.) as previously described (Yuan et al., 2017; Luo et al., 2018). At the end of the infusion, the pipette was kept *in situ* for at least 5 min and then withdrawn slowly. After injections, the mice used for *in vivo* fiber photometry experiments were implanted with an optical fiber cannula (Fiber core 200 μm, 0.37 numerical aperture (NA), Newdoon, China) into the dorsal striatum. The fiber cannula was implanted 0.2 mm above the virus injection site. After injection and implantation, the mice were placed on a heating pad for post-operative recovery. Mice were housed for at least 2 weeks after injections for complete recovery and to allow viral expression prior to any experiments.

### Electroencephalogram/Electromyogram (EEG/EMG) Electrode Implantation

As previously described (Yuan et al., 2017), the EEG/EMG electrode consists of two stainless steel screws with wire leads for EEG recording and two Teflon-coated stainless-steel wires (Cooner Wire, United States) for EMG recording. To implant the electrode, two small craniotomy holes were made in the frontal (AP: +1.5 mm, ML: -0.7 mm) and parietal (AP: -1.5 mm, ML: -1.0 mm) regions with a cranial drill. The EEG electrodes were screwed into the craniotomy holes and the EMG wires were bilaterally placed into the trapezius muscles. All the electrodes were attached to a mini-connector and fixed to the skull with dental cement.

### Polysomnographic Recording and Analysis

After 2 weeks of post-operative recovery, each animal was connected to an EEG/EMG recording cable in a recording apparatus (transparent barrel) and habituated for 3 days before polysomnographic recordings were conducted. The uninterrupted, synchronous recordings of EEG and EMG were performed by means of a slip ring, which was designed to let the mice move freely. Cortical EEG and neck EMG signals were amplified and filtered (Biotex Kyoto, Japan. EEG, 0.5–30 Hz; EMG, 20–200 Hz), digitized at a sampling rate of 512 Hz, and recorded by a Power 1401 digitizer and Spike2 software (CED, Cambridge, United Kingdom). The Spike2 data were then converted to text format for the analysis of vigilance states using SleepSign software (Kissei Comtec, Nagano, Japan). After the experiment was completed, the EEG/EMG data were automatically classified off-line using 4-s epochs for wakefulness, REM sleep, and NREM sleep using SleepSign software according to standard criteria (Huang et al., 2005). These automatically defined classifications were checked manually and corrected if necessary. Wakefulness was defined as periods of desynchronized, low-amplitude EEG and heightened EMG activity with phasic bursts; NREM sleep was defined as periods of synchronized, high-amplitude, low-frequency (delta band: 0.5–4 Hz) EEG and low EMG activity (compared with wakefulness) without phasic bursts; REM sleep was defined as periods with a pronounced theta rhythm (6–10 Hz) and no EMG activity.

### Fiber Photometry

Following the 2-week recovery period from the virus injection and implantation surgery, dLight1.1 fluorescence emission was recorded with a fiber photometry system (Thinkerbiotech, Nanjing, China) using methods similar to previous studies (Li et al., 2016; Luo et al., 2018). The fiber photometry was performed at 8:00–18:00. Briefly, to record fluorescent signals, the beam from a 488-nm laser (OBIS 488LS, Coherent, United States) was reflected by a dichroic mirror (MD498; Thorlabs), focused by a 10× objective lens (NA = 0.3, Olympus), and then coupled to an optical commutator (Doric Lenses, Canada). An optical fiber (230 mm optical density [O.D.], NA = 0.37, 1 m long) guided the light between the commutator and the implanted optical fiber. The laser power was adjusted at the tip of the optical fiber to a low level of 10–20 μW, to minimize bleaching. The dLight1.1 fluorescent signal was bandpass-filtered (MF525-39, Thorlabs) and collected by a photomultiplier tube (R3896, Hamamatsu). An amplifier (C7319, Hamamatsu) was used to convert the photomultiplier tube current output into voltage signals, which was further filtered through a low-pass filter (40 Hz cut-off; Brownlee 440). The photometry analog voltage signals were digitalized at 512 Hz and recorded by a Power 1401 digitizer and Spike2 software (CED, Cambridge, United Kingdom) simultaneously with polysomnographic recordings.

Photometry data were analyzed by customized Matlab software (Matlab, 2016a, MathWorks, United States) as described in our previous study (Luo et al., 2018). In brief, the photometry data were exported from Spike2 software in Matlab format for further analysis. The signal data were smoothed with a moving average filter (0.2 s span). For each session, the photometry signal F was converted to Δ*F*/*F* by calculating Δ*F*/*F* = (*F* -*F*_mean_)/*F*_mean_, where *F*_mean_ is the average fluorescence in recording episode. We recorded data for 4–10 h per session and calculated the averaged Δ*F*/*F* during periods of wakefulness, NREM, and REM sleep. For the analysis sleep-state transitions, we identified each state transition and aligned Δ*F*/*F* with a ±60 s window before and after the switch point. For stimuli analysis, the photometry signal was aligned with a ±20 s window before and after the event onset. For the modafinil, caffeine, and cage-change experiments, we recorded signals for 6 h (from 1 h before to 5 h after the administration of drugs or the cage change) and calculated the averaged Δ*F*/*F* value pre- and post-treatment.

### Auditory Tone and Door-Opening Test

To examine whether striatal dopamine levels respond to external stimuli, a high-frequency auditory tonal stimulus (70 dB, 2–4 kHz, 10 s duration) or a sudden door-opening stimulus was applied to mice during NREM sleep or wake periods as previously described (Cho et al., 2017). The loudspeaker was placed on top of the recording cage about 50 cm above the mouse and the intensity of the auditory tone inside the cage was calibrated with a sound meter (Uni-Trend UT350, Dongguan, China). The auditory stimulus and door-opening were both performed suddenly (without warning) when the mouse was either asleep or awake. Then the mice were allowed to rest without disturbance for 5–10 min before the next stimulation. Each type of stimulus was repeated at least three times for each mouse.

### Pharmacological Treatments

One hour following the onset of the photometry recording, modafinil (Sigma-Aldrich, United States) was dissolved in sterile saline containing 10% DMSO and 2% (w/v) cremophor and administered intraperitoneally at doses of 45 and 90 mg/kg. Caffeine (Alfa Aesar, United Kingdom) was dissolved in sterile saline and given intraperitoneally at a dose of 15 mg/kg. Both drugs were prepared fresh, immediately before use.

### Histology

Histological verification of viral expression was performed as described previously (Luo et al., 2018). After all the experiments were completed, the mice were deeply anesthetized with an overdose of pentobarbital and transcardially perfused with phosphate-buffered saline (PBS) followed by 4% paraformaldehyde (PFA) in PBS. Then, the brains were post-fixed in 4% PFA in 0.1 M phosphate buffer (PB; pH 7.4) for 6 h. Next, the brains were then transferred to 20% sucrose in PBS until they sank to the bottom, followed by an incubation in 30% sucrose until they sank to the bottom. Then, the tissue was embedded in optimum cutting temperature (OCT) compound, frozen, and coronal sections were cut at 30 μm by a freezing microtome (Leica, Germany). Since dLight1.1 cannot be detected directly by native fluorescence, a further immunohistochemistry was required (Patriarchi et al., 2018). The brain slices were washed in PBS and incubated in chicken anti-GFP primary antibody (1:5000 dilution; GFP-1020, Aves Labs, United States) at 4°C overnight. The next day, the sections were incubated in Alexa Fluor 488-conjugated donkey anti-chicken secondary antibody (1:1000 dilution, Cat. # 703-545-155, Jackson ImmunoResearch, United States) for 2 h at room temperature. Finally, slices were washed in PBS and mounted on glass slides using DAPI Fluoromount-G (Southern Biotech, Cat. # 0100–20). Images were captured by a fluorescence microscope (IX71, Olympus).

### Statistical Analyses

Data are presented as the means ± SEM. Paired or unpaired Student’s *t*-tests were used for two-group comparisons and one-way analysis of variance (ANOVA) was used for multiple-group comparisons. A two-way ANOVA was used to analyze the experiments with modafinil and caffeine treatment. Following the ANOVA, Sidak or Bonferroni’s *post hoc* tests were used to make pairwise comparisons. All the statistical tests were two-tailed and *P*-values less than 0.05 were considered significant. All the statistical analyses were performed using Prism 7.0 (GraphPad Software, United States) and MATLAB R2016a software.

## Results

### Striatal Dopamine Levels Across Spontaneous Sleep–Wake Cycle

**FIGURE 1:**
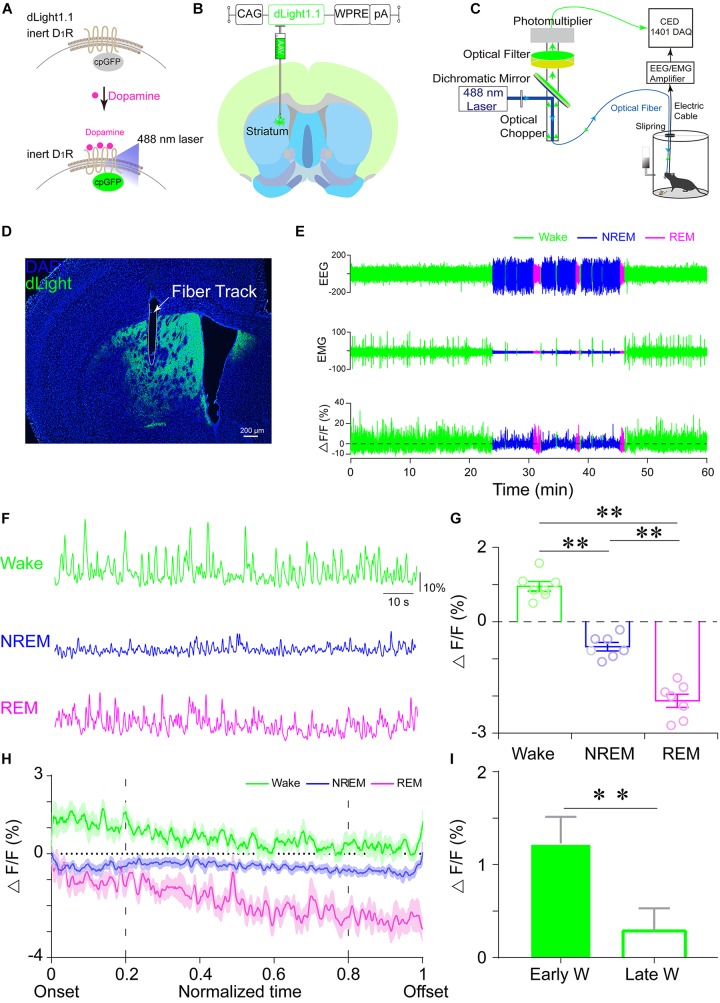
Striatal dLight1.1 signals at distinct spontaneous sleep–wake states. **(A)** Schematic diagram of dLight1.1 with the dopamine D_1_ receptor and circularly permuted GFP (cpGFP) module (upper panel) and the working principle of dLight1.1. **(B)** Schematic showing the injection of AAV-CAG-dLight1.1 into the dorsal striatum. **(C)** Schematic showing the setup for fiber photometry used to assess dLight1.1 fluorescence with simultaneous polysomnographic recordings. **(D)** Expression of dLight1.1 in the dorsal striatum. The scale bar is 200 μm. **(E,F)** Representative EEG, EMG, and fluorescent photometry signal traces of striatal dLight1.1. during distinct sleep–wake states (green, wake; blue, NREM sleep; magenta, REM sleep). **(G)** Quantification of the average striatal dLight1.1 signal at distinct sleep–wake states. One-way ANOVA: *F*_2,18_ = 116.1, *P* < 0.0001; Tukey’s *post hoc* test: wake vs. NREM sleep ^∗∗^*P* < 0.0001, wake vs. REM sleep ^∗∗^*P* < 0.0001, NREM vs. REM sleep ^∗∗^*P* < 0.0001; *n* = 7 mice. **(H)** Temporal dynamics of the striatal dLight1.1 signal during long-term wake (green), NREM sleep (blue), and REM sleep (magenta) episodes within normalized time. **(I)** Striatal dLight1.1 signal at the early wake period (first 20% of wake period) and the late wake period (last 20% of wake period) (*t*_6_ = 5.058, ^∗∗^*P* = 0.0023, *n* = 7 mice).

Patriarchi et al. (2018) showed that the dLight1.1 plasmid was silent in the absence of dopamine. When dopamine is released from presynaptic terminals, it binds to the dLight1.1 sensor and dramatically increases its fluorescence (Figure 1A). In order to ensure the efficient and precise expression of dLight1.1 in the dorsal striatum, an AAV encoding dLight1.1 under the control of a CAG promoter was unilaterally injected into the dorsal striatum of mice (Figure 1B). The mice were also chronically implanted with (1) a fiberoptic probe upon the virus-injection site for subsequent delivery of light excitation and collection of dLight1.1 fluorescence and (2) EEG/EMG electrodes for simultaneous polysomnographic recordings (Figure 1C). The success of the virus infection and the appropriate location of the fiberoptic implant were verified in each mouse after all experiments were completed. As shown in Figure 1D and Supplementary Figure S1), dLight1.1 fluorescence was robustly expressed at the injection site in the dorsal striatum.

**FIGURE 2:**
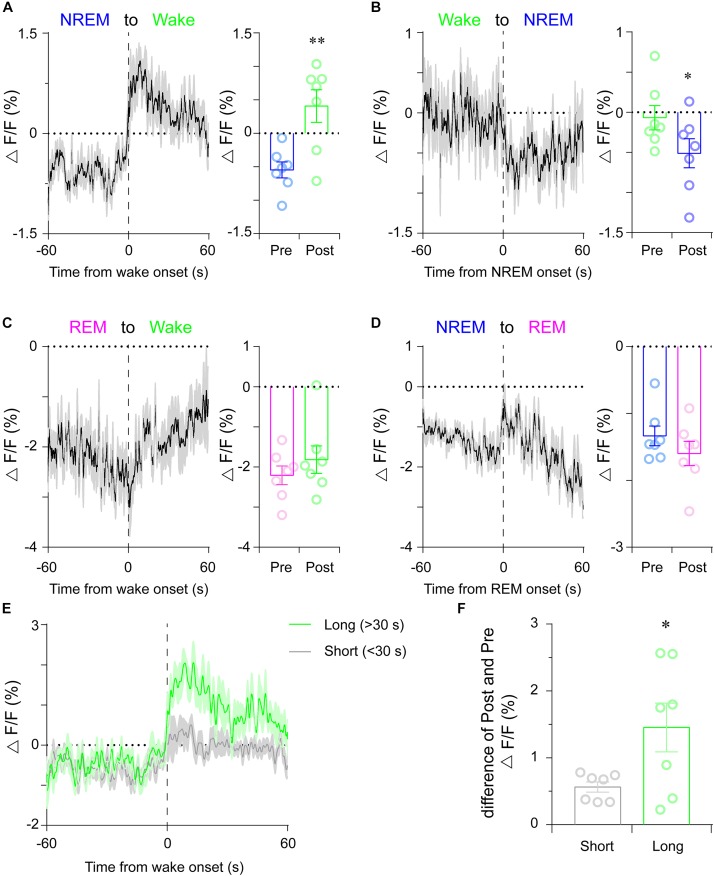
Striatal dLight1.1 signal dynamics across spontaneous sleep-state transitions. **(A)** Striatal dLight1.1 signals across NREM sleep-to-wake transition. (Left) The time course of the dLight1.1 signal across the NREM sleep-to-wake transition. (Right) The average amplitude of the dLight1.1 signal 60 s pre- and post-transition (*t*_6_ = 5.441, ^∗∗^*P* < 0.01, *n* = 7 mice). **(B)** Striatal dLight1.1 signal at the wake-to-NREM sleep transition. (Left) The time course of the dLight1.1 signal across the wake-to-NREM sleep transition. (Right) The average amplitude of the dLight1.1 signal 60 s pre- and post-transition (*t*_6_ = 2.528, ^∗^*P* = 0.044). **(C)** Striatal dLight1.1 signal at the REM sleep-to-wake transition. (Left) The time course of the dLight1.1 signal across the REM sleep-to-wake transition. (Right) The average amplitude of the dLight1.1 signal 60 s pre- and post-transition the (*t*_6_ = 1.053, *P* = 0.333). **(D)** Striatal dLight1.1 signal at the NREM-to-REM sleep transition. (Left) The time course of the dLight1.1 signal across the NREM-to-REM sleep transition. (Right) The average amplitude of the dLight1.1 signal 60 s pre- and post-transition (*t*_6_ = 1.509, *P* = 0.182). **(E)** Time courses of the striatal dLight1.1 signals across NREM sleep-to-long wake or NREM sleep-to-short wake periods. **(F)** The change in the dLight1.1 signal after long or short wake periods compared with the 60 s pre-wake period (*t*_12_ = 2.388, ^∗^*P* = 0.034). Data are presented as the mean (black trace) ± SEM (gray shading) in **(A–D)** and as the mean (long wake period, green; short wake period, gray) ± SEM (shading) in **(E)**.

To examine whether the striatal dopamine levels correlated with distinct vigilance states, we recorded striatal dLight1.1 fluorescent signals across spontaneous sleep–wake cycle. As shown in Figure 1E,F, the fluctuations of dLight1.1 fluorescence were correlated with the EEG/EMG signals. To compare the dLight1.1 signal amplitude during distinct vigilance states, the fluorescent signals were averaged in a state-dependent manner. We found that the mean striatal dLight1.1 signal was significantly higher during wakefulness (0.952% ± 0.128%) than during NREM sleep (-0.6% ± 0.114%) or REM sleep (-2.129% ± 0.179%), which exhibited the lowest fluorescence (Figure 1G; *n* = 7 mice; *F*_2,18_ = 116.1, *P* < 0.01; *post hoc* Tukey test: wake vs. NREM *P* < 0.01, wake vs. REM *P* < 0.01, NREM vs. REM *P* < 0.01). Although the peak value of REM sleep is higher and the trough value is lower (Supplementary Figure S3), the mean fluorescent signal is the lowest in REM sleep. Moreover, Dahan et al. (2007) found that the dopamine neuronal activity during REM sleep showed a pronounced bursting pattern with decreased amplitude. This firing pattern of dopaminergic neurons may be the reason that striatal dopamine level during REM sleep was more divergent with higher peak and lower trough. To examine the temporal dynamics of striatal dopamine during long-term sleep–wake states (duration longer than 30 s), we normalized the variable duration of sleep–wake states to a unit-less time window from 0 (state onset) to 1 (state offset). During the wakefulness period, dLight1.1 fluorescence peaked soon after the onset of wakefulness and gradually attenuated (Figure 1H). We calculated the mean fluorescence during the first 20% and the last 20% of the wake episode. We found that dLight1.1 fluorescence in the early 20% of the wake episode was significantly higher than that in the late 20% (Figure 1I; *n* = 7 mice; *t* = 5.058, *P* = 0.0023). These findings demonstrated that striatal dopamine levels not only varied across spontaneous sleep–wake states but also showed dynamic changes within wakefulness episodes. Next, we assessed the striatal dopamine levels during state transitions. We found that the striatal dLight1.1 signal increased significantly during NREM sleep-to-wake transitions (Figure 2A; *t* = 5.441, *P* < 0.01), whereas it decreased during wake-to-NREM sleep transitions (Figure 2B; *t* = 2.528, *P* = 0.044). However, there was no significant dLight1.1 fluorescence change during REM sleep-to-wake transitions and NREM-to-REM sleep transitions (Figure 2C, *P* = 0.333 and Figure 2D, *P* = 0.182). Since the diversity of animal behaviors depend on the duration of the wakefulness episode, we further examined whether striatal dopamine levels fluctuated with the duration of wakefulness episodes; we calculated the dLight1.1 signal at longer wake episodes (duration > 30 s) and brief wake episodes (duration < 30 s). Interestingly, the net growth of dLight1.1 fluorescence was significantly higher when mice were awake for longer periods than for brief wake periods (Figure 2E,F; *t* = 2.388, *P* = 0.0343). These results indicated that striatal dopamine levels at wake onset were correlated with the duration of the following wake episode. Taken together, these findings demonstrated that striatal dopamine levels were highest during wakefulness and that they fluctuate dynamically across spontaneous state transitions.

### Striatal Dopamine Levels in Response to Acute External Stimuli

**FIGURE 3:**
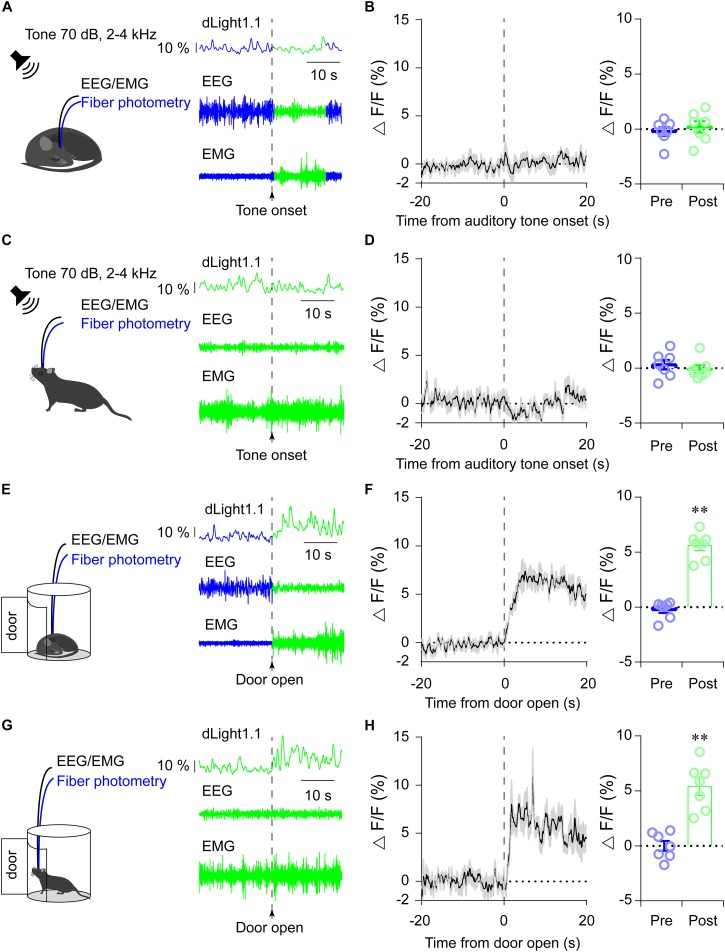
Striatal dLight1.1 fluorescence in response to acute stimuli. **(A**, Left) Schematic showing high-frequency auditory tones applied in the sleep state simultaneously with fiber photometry and EEG/EMG recording. (Right) Example traces of the fluorescence signal, EEG, and EMG before and after the onset of the auditory tone during sleep. **(B**, Left) The time course of the striatal dLight1.1 signal in response to auditory tones when mice were sleeping. (Right) Average fluorescence before and after onset of the auditory tone (*t*_6_ = 1.243, *P* = 0.260). **(C**, Left) Schematic showing auditory tones applied in the awake state simultaneously with fiber photometry and EEG/EMG recording. (Right) Example traces of the fluorescence signal, EEG, and EMG before and after the onset of the auditory tone during the awake state. **(D**, Left) The time course of the striatal dLight1.1 signal in response to auditory tones when mice were awake. (Right) Average fluorescence before and after onset of the auditory tone (*t*_6_ = 1.771, *P* = 0.127). **(E**, Left) Schematic showing the sudden opening of the recording-cage door during the sleep state. (Right) Typical traces of the fluorescence signal, EEG, and EMG before and after the onset of the door-opening stimulus during sleep. **(F**, Left) The time course of the striatal dLight1.1 signal in response to the door-opening stimulus while the mice were sleeping. (Right) Average fluorescence before and after onset of the door-opening stimulus (*t*_6_ = 12.15, ^∗∗^*P* < 0.0001). **(G**, Left) Schematic showing the sudden opening of the recording-cage door while mice were in the awake state. (Right) Typical traces of the fluorescence signal, EEG, and EMG before and after the onset of the door-opening stimulus during the wake period. **(H**, Left) The time course of the striatal dLight1.1 signal in response to the door-opening stimulus while the mice were awake. (Right) Average fluorescence before and after onset of the door-opening stimulus (*t*_6_ = 11.18, ^∗∗^*P* < 0.0001).

To investigate the dynamics of dopamine levels in the dorsal striatum in response to external stimuli, we recorded striatal dLight1.1 fluorescence while simultaneously conducting polysomnographic recordings in animals exposed to diverse salient stimuli and stimulants. The unexpected presentation of an auditory tone stimulus (70 dB, 2–4 kHz, 10 s duration) was employed as previously described (Cho et al., 2017). We exposed the mice to an auditory tone while asleep or awake (Figure 3A,C) and observed that the mice were immediately awakened when the tone was applied during the sleep period (Figure 3A). However, there were no detectable changes in striatal dLight1.1 fluorescence in response to the auditory stimulus either when mice were asleep or awake (Figure 3B: *P* = 0.260 and Figure 3D: *P* = 0.127). These results indicated that acute, short auditory tone stimuli did not elevate striatal dopamine release. Surprisingly, the striatal dLight1.1 signal ascended when the door of the mouse’s home cage was suddenly opened at the end of the trial session. This observation prompted us to systematically investigate whether unexpected door-opening during sleep or wake states induced striatal dLight1.1 signal changes. We discovered that the striatal dLight1.1 signal rapidly increased whenever the home-cage door was opened suddenly during the sleep period (Figure 3E,F; *t* = 12.15, *P* < 0.01) and the wake period (Figure 3G,H; *t* = 11.18, *P* < 0.01). In addition, the amplitude of the striatal dLight1.1 signal induced by sudden door-opening was higher than the amplitude during spontaneous awake periods. Although both auditory tone stimulation and door-opening were able to wake up sleeping mice, the door-opening stimulus elevated dopamine release while auditory stimulation did not. The behavioral paradigm door-opening test maybe mix with visual and olfactory stimuli. In order to explore whether visual and olfactory stimuli enhance striatal dopamine tone, predator odor TMT or light flash were employed. We used light flash at 1 Hz for 10 s when mice were sleeping or awaking (Supplementary Figure S2A), and found that flash during awaking increased striatal dLight1.1 fluorescence (Supplementary Figure S2C, *t* = 4.486, *P* = 0.0463), but failed to enhance during sleep (Supplementary Figure S2B, *t* = 0.9181, *P* = 0.4555). These results indicated that awareness of visual stimuli enhanced the striatal dopamine tone. Application of air or predator odor TMT didn’t enhance striatal dLight1.1 fluorescence (air, *t* = 0.4803, *P* = 0.6784; TMT, *t* = 0.2858, *P* = 0.8019. Supplementary Figures S2D–F). These results indicated that striatal dopamine levels responded only to specific acute stimuli.

### Striatal Dopamine Levels in Response to Cage Change

**FIGURE 4:**
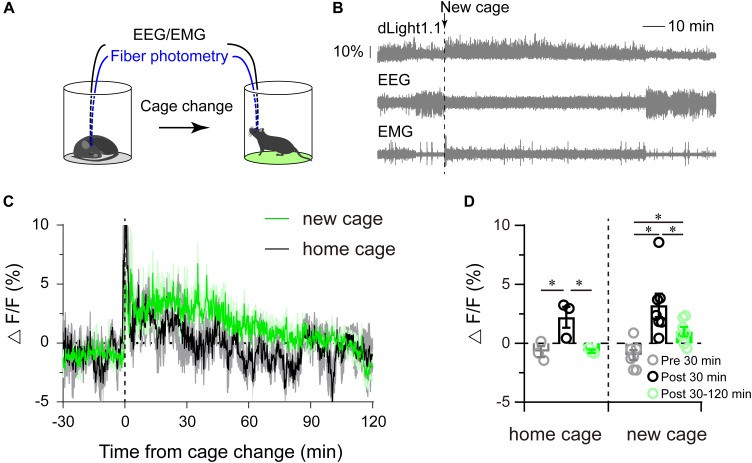
Striatal dLight1.1 fluorescent signal in response to a new environment. **(A)** Schematic showing the cage-change procedure, where mice were moved to a new cage. **(B)** A typical example of the fluorescence signal, EEG, and EMG traces before and after the cage-change. **(C)** Time course of the striatal dLight1.1 signal in response to the cage-change, (black is to home cage, green is to new cage). **(D)** Average striatal dLight1.1 signal 30 min before, 30 min and 30–120 after the cage change to home cage or new cage (one-way ANOVA, home cage: *F*_2,4_ = 7.284, *P* = 0.0464; new cage: *F*_2,12_ = 10.1832, *P* = 0.0026. post comparisons followed by PLSD).

Our previous study showed that dopamine receptors were necessary for arousal when mice were exposed to new environments (Qu et al., 2010; Xu et al., 2014). However, whether exposure to a new environment augmented the striatal dopamine level was still unclear. To address this question, the cage-change model was employed to mimic a new environment (Figure 4A). We found that mice exhibited continuous wakefulness for almost 2 h after tail handled followed by moving to a new cage (Figure 4B), while mice kept awake for about 30 min after tail handled followed by returning to their home cages, coincident with our previous results (Qu et al., 2010; Xu et al., 2014). Striatal dLight1.1 fluorescence sharply increased when the mice were tail handled, then gradually attenuated to baseline about 2 h after moved to a new cage, whereas quickly decreased to baseline about 30 min after returned to home cages after tail handled (Figure 4B,C). We calculated the mean fluorescence 30 min before (serving as the baseline), 30 min and 30–120 min after returned home cage or moved to a new cage, and found that the mean striatal dLight1.1 fluorescence was significantly higher than baseline for post 30 min when mice were tail handled followed by returned to home cage, and but there was no statistical significance between post 30–120 min and baseline (Figure 4D. *F*_2,4_ = 7.284, *P* = 0.0464), However, when mice were tail handled followed by moved to new cage, the mean striatal dLight1.1 fluorescence was significantly higher for post 30 min and the following 90 min than baseline (Figure 4D. *F*_2,12_ = 10.1832, *P* = 0.0026). These findings revealed that moving the mice to home cage or new cage induced wakefulness and enhanced striatal dopamine release which sustained for 30 min for mice returned to home cage and for at least 2 h in mice exposed to a new cage.

### Stimulants Induced Striatal Dopamine Release

**FIGURE 5:**
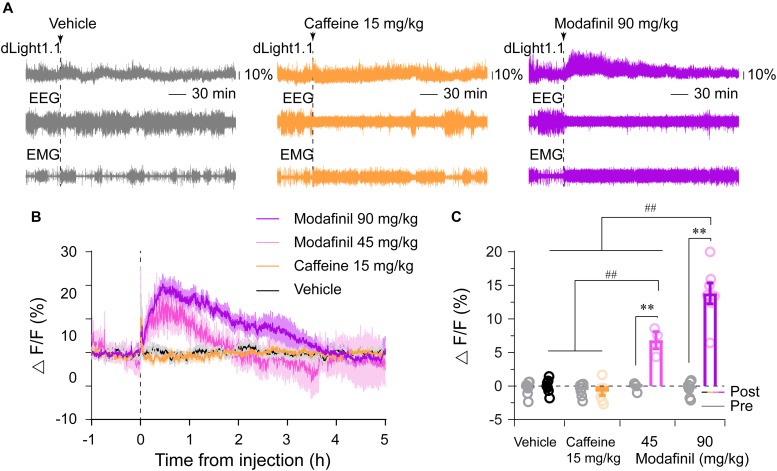
Effects of caffeine (15 mg/kg) and modafinil (45 and 90 mg/kg) on the striatal dLight1.1 fluorescent signal. **(A)** Typical examples of the striatal dLight1.1 fluorescent signal, EEG, and EMG traces following vehicle (10% DMSO), caffeine, and modafinil administration. **(B)** Time courses of the striatal dLight1.1 fluorescent signal following vehicle, caffeine, and modafinil administration. **(C)** Average striatal dLight1.1 fluorescent signal in the 1 h before (gray circle and bar) and 2 h after each administration. Two-way ANOVA between time and stimuli: *F*_1,20_ (time) = 147.5142, *P* < 0.0001, pre–post comparisons followed by Bonferroni’s test: ^∗∗^*P* < 0.0001; *F*_3,20_ (stimuli) = 29.5910, *P* < 0.0001. Stimuli comparisons followed by Sidak’s test: ^##^*P* < 0.0001).

Stimulants such as caffeine and modafinil are universally used to stay awake and to boost mental performance. Our previous studies have shown that caffeine promoted wakefulness via adenosine A_2A_ receptors (A_2A_Rs) (Huang et al., 2005; Lazarus et al., 2011), whereas modafinil induced wakefulness via D_1_Rs and D_2_Rs (Qu et al., 2008). A_2A_Rs were reported to be densely co-expressed with D_2_Rs in the dorsal striatum (Svenningsson et al., 1999; Lazarus et al., 2012, 2013). however, it is still unknown whether modafinil and caffeine alter striatal dopamine levels. We found that caffeine (15 mg/kg) or modafinil (45, 90 mg/kg) promoted continuous wakefulness for about 2, 3, or 5 h, respectively (Figure 5A), consistent with our previous results (Huang et al., 2005; Qu et al., 2008). Administration of modafinil strongly increased striatal dLight1.1 fluorescence, with dLight1.1 signals rapidly reaching a peak and then gradually attenuating (Figure 5A,B). For better comparison of the effects of each drug on striatal dopamine tone, we calculated the average fluorescence 1 h before (serving as the baseline) and over the 2 h after each administration. We chose 2 h for comparisons because caffeine 15 mg/kg induced wakefulness for 2 h, although modafinil promoted longer effects. As shown in Figure 5C, administration of modafinil significantly enhanced the striatal dLight1.1 fluorescence, but vehicle and caffeine didn’t, compared with their respective baseline (*F*_1,20_ = 147.5142, *P* < 0.0001, pre–post comparisons followed by Bonferroni’s test: modafinil 90 mg/kg vs. baseline, *P* < 0.0001; modafinil 45 mg/kg vs. baseline, *P* < 0.0001; caffeine vs. baseline, *P* > 0.9999; vehicle vs. baseline, *P* > 0.9999). Moreover, the mean dLight1.1 signals for 2 h after administration of modafinil 90 mg/kg was 13.81% ± 1.55%, significantly higher than 6.83% ± 1.27% for modafinil 45 mg/kg, -0.86% ± 0.55% for caffeine 15 mg/kg, and -0.02% ± 0.40% for vehicle. The dLight signal for modafinil at 45 mg/kg was higher than that for caffeine 15 mg/kg or vehicle, but there was no statistical significance between caffeine 15 mg/kg and vehicle (*F*_3,20_ = 29.5910, *P* < 0.0001. Comparisons followed by Sidak’s test: modafinil 90 mg/kg vs. modafinil 45 mg/kg, *P* < 0.0001; modafinil 45 mg/kg vs. caffeine, *P* < 0.0001; caffeine vs. vehicle, *P* = 0.4198). Taken together, these findings indicated that modafinil increased striatal dopamine levels but caffeine did not.

## Discussion

Using a dopamine sensor and simultaneous polysomnographic recordings, we demonstrated that striatal dopamine levels were highest during wakefulness and dopamine fluctuations correlated with spontaneous sleep–wake transitions. Furthermore, we revealed that some external salient stimuli and certain wake-promoting stimulants elicited striatal dopamine release. These findings provide strong evidence that dopamine in the dorsal striatum is important for wakefulness under baseline conditions, induced by cage change or wake-promoting drug modafinil but not caffeine.

The dorsal striatum receives robust dopaminergic inputs from the SNc, as well as some input from the VTA and DRN (Beckstead et al., 1979; Stratford and Wirtshafter, 1990; Poulin et al., 2018). Although the single-unit firing rate of SNc and VTA dopaminergic neurons in cats and rats shows no changes across the stages of sleep or waking (Trulson et al., 1981; Miller et al., 1983; Steinfels et al., 1983), the specific enhancement of VTA and SNc dopaminergic neuron activity by optogenetic or chemogenetic approaches dramatically induces wakefulness (Eban-Rothschild et al., 2016; Oishi et al., 2017a; Yang et al., 2018). Furthermore, our recent work demonstrates that the inhibition of striatal D_2_R/A_2A_R-containing neurons, mimicking the action of dopamine on D_2_Rs, promotes wakefulness (Yuan et al., 2017). Axonal dopamine release not only depends on the firing rate and pattern of dopaminergic neurons but also on the concentration of calcium (Ca^2+^) (Kawagoe et al., 1992; Chen et al., 2011). Moreover, dopamine release is assumed to reflect a global response to the activity of midbrain dopaminergic neurons at a population level (Rice et al., 2011). Recent photometry data demonstrate that the population-level calcium signal of VTA and DRN dopaminergic neurons are correlated with the sleep–wake cycle (Eban-Rothschild et al., 2016; Cho et al., 2017). Using intracerebral microdialysis, Lena et al. (2005) elaborated that the dopamine concentrations in the NAc, downstream of the VTA, are higher during both wakefulness and REM sleep compared with NREM sleep in rats. Trulson (1985) used voltammetry to measure the release of dopamine in the dorsal striatum of cats across their sleep–wake cycle at a 5-min temporal resolution. During the 45-min recording consisting of consecutive 15-min periods of each sleep stage, the striatal dopamine voltammetric peak decrease from wake to NREM sleep, and from NREM to REM sleep in cats. Consistent with this finding, our present study showed that striatal dopamine levels were at their highest during wakefulness and their lowest during REM sleep. Taking advantage of the high temporal resolution of dLight1.1, we further analyzed the dynamic variation of striatal dopamine during short-term state transitions and found that striatal dopamine increased significantly during NREM sleep-to-wake transitions and decreased during wake-to-NREM sleep transitions. Moreover, extracellular dopamine levels in mice striatal slices were reported to oscillate across the light/dark cycle (Ferris et al., 2014). These pieces of evidence suggest that recording the electrophysiological activity of single dopaminergic neurons does not accurately reflect the functional state of the central dopaminergic system. Collectively, the above findings solidly demonstrate that striatal dopamine release correlates with the sleep–wake cycle, despite the fact that dopaminergic neuron firing is uncorrelated with vigilance states.

The dopamine level in the dorsal striatum is a signal for prolonged time in wakefulness and crucial for the maintenance of wakefulness. Chemogenetic or optogenetic activation of SNc, VTA, or DRN dopaminergic neurons induce a long-lasting period of wakefulness (Eban-Rothschild et al., 2016; Cho et al., 2017; Oishi et al., 2017a; Yang et al., 2018). The fluctuation in DRN dopaminergic activity across sleep-to-wake transitions is significantly larger when mice are awake for a longer period than when they are briefly awake (Cho et al., 2017). Consistent with this, our present study showed that striatal dopamine levels were higher when mice had longer periods of wakefulness (>30 s) than when they had brief periods of wakefulness (<30 s). These findings suggest that the striatal dopamine level can predict the length of the following wake episode. Chemogenetic inhibition of SNc or VTA dopaminergic neurons promotes sleep at the expense of wakefulness (Eban-Rothschild et al., 2016; Yang et al., 2018). Inhibition of dopaminergic neurons can, in theory, reduce striatal dopamine release and then lead to striatal D_1_R/D_2_R inactivation. Our previous study revealed that the genetic deletion of D_2_Rs destabilizes the wake stage and shortens the duration of wakefulness episodes (Qu et al., 2010). What’s more, chemogenetic activation of D_2_Rs-containing neurons in the dorsal striatum promoted sleep (Yuan et al., 2017). Our present study showed that striatal dopamine levels peaked soon after wake onset and gradually reduced during the wake period. These pieces of evidence collectively suggest that decreasing dopamine levels can facilitate sleep initiation and lessens alertness. The multiplicity of arousal systems guarantees diverse behaviors in the normal individual. It has been reported that histaminergic tuberomammillary neurons are crucial for brief wakefulness (Huang et al., 2006). We can conclude from the literature that distinct arousal mechanisms govern different levels or types of alertness.

Our study showed that striatal dopamine levels not only correlated with the spontaneous sleep–wake cycle, but also responded to salient environmental stimuli. In addition to homeostatic and circadian drives as well as emotion, a good sleep also requires a quiet and safe environment (Saper et al., 2005). An unexpected sound or predator invasion can disrupt the quality of sleep (Saper et al., 2005; Eban-Rothschild et al., 2016; Cho et al., 2017). Consistently, the acute auditory tone and sudden door-opening of the home cage immediately awoke mice from NREM sleep. We found that door-opening and light flash induced striatal dopamine release, whereas the auditory tone failed to do so. An early study found that opening the door of a cat’s housing chamber or the presence of the experimenter in the cat’s field of vision is associated with the bursting activity of single-unit SNc dopaminergic neurons (Steinfels et al., 1983). While dopaminergic neurons fire in a slow, irregular fashion under baseline conditions, resulting in a tonic release of dopamine, they fire in bursts in response to salient environmental stimuli, which lead to phasic increases in dopamine release (Overton and Clark, 1997). Consistent with this, our data showed that more the striatal dopamine was released following exposure to salient stimuli than during spontaneous wakefulness. Mapping the inputs to midbrain dopaminergic neurons may help us understand their different responses to auditory and visual stimuli. Monosynaptic tracing studies demonstrate that SNc and VTA dopaminergic neurons receive dense input from the superior colliculus, a key structure processing visual information, but received hardly any input from the auditory system (Watabe-Uchida et al., 2012; Lerner et al., 2015). Moreover, previous studies have demonstrated that the superior colliculus is necessary to relay short-latency visual information to dopamine-containing regions of the ventral midbrain in rats (Comoli et al., 2003; Dommett et al., 2005). The door-opening stimulus combined both auditory and visual stimuli, making it sufficient to elicit striatal dopamine release. However, an auditory tone as a conditioned stimulus combined with a reward unconditioned stimulus induces a large dopamine release upon repeated cue-reward pairing but not in the first training session (Patriarchi et al., 2018). Collectively, the above findings suggest that striatal dopamine responds to specific stimuli.

Cage-change is a mouse model that mimics the human first-night effect, which can be observed in unfamiliar sleeping environments. We previously found that the genetic deletion or pharmacological blockade of D_2_Rs (densely expressed in the dorsal striatum) reduce the duration of wake episodes in mice following being moved to a new cage. The plasma corticosterone levels are elevated after cage change, suggesting that cage change or new environment induces an elevating arousal level (Qu et al., 2010; Xu et al., 2014). Eban-Rothschild et al. (2016) found that transferring the mice to a new environment or introducing novel objects to their home space enhance calcium activity in VTA dopaminergic neurons. Moreover, chemogenetic inhibition of VTA dopaminergic neurons prompts nest-building behavior and promotes sleep. Consistently, we found that cage change induced a significant increase in striatal dopaminergic tone. Therefore, we supposed that cage change induced an elevating arousal level with more increases in the striatal dopaminergic tone, suggesting that dopamine tone may relate to arousal level and the dopaminergic system may be a target for treating insomnia caused by environmental stimuli.

In the present study, we employed auditory tone, door-opening, light flash, predator odor, and cage change paradigm combined with striatal dopamine tone recording and found that striatal dopamine responds to specific stimuli. Striatal dopamine activities also are associated with lots of behaviors, such as locomotion, motivation, reward, and stress, all of which operate on the basis of wakefulness. The role of dopamine in motor behavior is extensively concerned. Rapid phasic signal in striatum-targeting dopaminergic axons is associated with triggering and locomotion in mice (Howe and Dombeck, 2016). Large proportion of SNc dopaminergic neurons transiently increased their activities before self-paced movement initiation in mice (da Silva et al., 2018). The activity of VTA dopaminergic neurons are increased during itch-induced scratching behavior in freely moving mice (Yuan et al., 2018). Dopamine is also involved in negative emotion. Intense exteroceptive stimuli, such as an electric shock on the tail or placing animals into an ice-water bath, provoke large and abrupt rises in the striatal dopamine signal (Keller et al., 1983). Dopamine neurons projecting to the anterior striatum display patterns of activity consistent with the reward value, while those projecting to the posterior tail of the striatum are activated by aversive and neutral stimuli, such as unexpected tone and air puff (Menegas et al., 2018). Taken together, striatal dopamine activities are associated with lots of behaviors, operating on the basis of wakefulness.

Modafinil is a wake-promoting drug used to treat daytime sleepiness. Numerous studies have suggested that modafinil promotes wakefulness by acting on the dopaminergic system Consistently, modafinil is found to bind dopamine uptake transporters (DATs) with low affinity (Mignot et al., 1994) and the deletion of the DAT gene in mice blocks the wake-promoting effects of modafinil (Wisor et al., 2001). We previously found that the blockade of D_1_Rs and D_2_Rs abolishes the arousal effects of modafinil (Qu et al., 2008). In addition, modafinil has been reported to enhance extracellular levels of dopamine in the NAc, PFC, and medial hypothalamus of rats (de Saint Hilaire et al., 2001; Murillo-Rodriguez et al., 2007). Moreover, optogenetic stimulation of dopaminergic terminals in the NAc and dorsal striatum induce wakefulness, whereas the same conditions in the PFC fail to induce wakefulness. This result suggests that the NAc and dorsal striatum could be specific targets of modafinil. In line with this, our present study found that modafinil robustly raised striatal dopamine levels. Another widely used stimulant, caffeine, is a psychoactive compound that is found to promote wakefulness via A_2A_Rs (Huang et al., 2005). A_2A_Rs are densely co-expressed with D_2_Rs in the striatum (Schiffmann et al., 1991). Previous study revealed that the genetic deletion of striatal A_2A_Rs abolishes arousal effect of caffeine (Huang et al., 2005; Lazarus et al., 2011). Chemogenetic inhibition of dorsal or ventral striatal A_2A_R positive neurons promote arousal, that mimic arousal effects of caffeine (Oishi et al., 2017b; Yuan et al., 2017). The external globus pallidus mediates the effect of dorsal striatal A_2A_R positive neurons on sleep, while ventral pallidum, but not VTA, mediates the effect of ventral striatal A_2A_R positive neurons on sleep. Our current study showed that caffeine did not enhance striatal dopamine levels. These results are consistent with previous studies that caffeine doesn’t increase the c-fos expression in the SNc (Bennett and Semba, 1998). The differential effects of modafinil and caffeine on striatal dopamine levels suggest that despite them both being wake-promoting compounds that target the basal ganglia, their arousal effects have different underlying mechanisms, dopaminergic system for modafinil and adenosine system for caffeine. Patients with PD suffer from severe EDS and nigrostriatal dopamine deficiency has been proposed to be responsible for PD (Adler and Thorpy, 2005). In fact, most PD therapeutic agents act by increasing dopaminergic activity. In this study, we found that modafinil dramatically elicited striatal dopamine release. Hence, we propose that modafinil may be a potential agent to treat EDS in PD patients with motor symptoms. In addition, the adenosine system, especially the A_2A_R, has emerged as an attractive non-dopaminergic target in the pursuit of improved therapy for PD (Antonini and Poewe, 2014). Our study showed that caffeine, a non-specific antagonist of adenosine receptors, did not increase striatal dopamine, suggesting that caffeine promotes arousal but does not depend on dopaminergic systems. Moreover, large clinic studies showed that caffeine or coffee consumption has been associated with a reduced risk of PD (Ross et al., 2000; Ascherio et al., 2001). Hence, we propose that caffeine or A_2A_R antagonism could be a prospective agent for EDS therapy in PD.

Pharmacological, genetic, and clinical studies have demonstrated that striatal dopamine is involved in numerous behavioral and psychological processes that operate on the basis of wakefulness, including motor behaviors, attention, motivation, reward, and feeding. Dysregulation of nigrostriatal dopamine results in severe neurological disorders such as PD and Huntington’s disease with diversified sleep disturbances. Our study demonstrated that striatal dopamine levels fluctuated across the spontaneous sleep–wake cycle and responded to external stimuli and wake-promoting stimulants. By understanding the dynamics of striatal dopamine under various conditions, our findings provide insight into the role of striatal dopamine in sleep regulation and suggest a potential treatment alternative for sleep disturbances in PD.

## Data Availability

All datasets generated for this study are included in the manuscript and/or the Supplementary Files.

## Author Contributions

HD, JW, Y-FY, W-MQ, and Z-LH: conceptualization. HD and Y-FY: methodology, investigation, and formal analysis. HD, Y-FY, YS, W-MQ, and Z-LH: writing original draft. HD, JW, Y-FY, YS, W-MQ, and Z-LH: revised the manuscript. W-MQ and Z-LH: supervision and funding acquisition.

## Conflict of Interest Statement

The authors declare that the research was conducted in the absence of any commercial or financial relationships that could be construed as a potential conflict of interest.

## Supplementary Material

FIGURE S1 Expression of dLight1.1 in the dorsal striatum in all mice except 2# which is in Figure 1D. The blue is DAPI, the green is dLight1.1. The scale bar is 200 μm.

FIGURE S2 Striatal dLight1.1 fluorescence in response to light flash and predator odor. **(A,D)** Schematic showing light flash **(A)** or predator odor **(D)** was applied simultaneously with fiber photometry. **(B)** Up panel: Example traces of the fluorescence signal, EEG, and EMG before and after the onset of the light flash during sleeping. Down panel: (Left) The time course of the striatal dLight1.1 signal in response to light flash when mice were sleeping. (Right) Average fluorescence before and after onset of the light flash (*t* = 0.9181, *P* = 0.4555). **(C)** Up panel: Example traces of the fluorescence signal, EEG, and EMG before and after the onset of the light flash during awake. Down panel: (Left) The time course of the striatal dLight1.1 signal in response to light flash when mice were awake. (Right) Average fluorescence before and after onset of the light flash (*t* = 4.486, *P* = 0.0463). **(E)** Up panel: example traces of the fluorescence signal, EEG, and EMG before and after the onset of air release during awake. Down panel: (Left) The time course of the striatal dLight1.1 signal in response to air delivery. (Right) Average fluorescence before and after onset of the air delivery (*t* = 0.4803, *P* = 0.6784). **(F)** Up panel: example traces of the fluorescence signal, EEG, and EMG before and after the onset of TMT delivery during awake. Down panel: (Left) The time course of the striatal dLight1.1 signal in response to TMT delivery. (Right) Average fluorescence before and after onset of the TMT delivery (*t* = 0.2858, *P* = 0.8019).

FIGURE S3 The distributions of dLight fluorescence values in each state. The red presents REM sleep, the blue for NREM sleep, and the green for wake. The fluorescence in REM sleep was more divergent, but more convergent in NREM sleep. There was 3.14% dLight fluorescence values in REM sleep higher than 0.1, 10.26% values higher than 0.05, 71.48% values lower than 0, and 7.51% values lower than -0.1, whereas there was 0.57% dLight fluorescence values in NREM sleep higher than 0.1, 4.42% values higher than 0.05, 62.18% values lower than 0, 0.04% values lower -0.1. Although it looks more higher in REM sleep because there a few higher values, the mean of dLight fluorescence was the lowest in REM sleep.
