# Supplementary figures and images for: Dorsal Striatum Dopamine Levels Fluctuate Across the Sleep–Wake Cycle and Respond to Salient Stimuli in Mice
Source: Front Neurosci. 2019 Mar 19;13:242. doi: 10.3389/fnins.2019.00242 (PMC6436203; doi:10.3389/fnins.2019.00242)

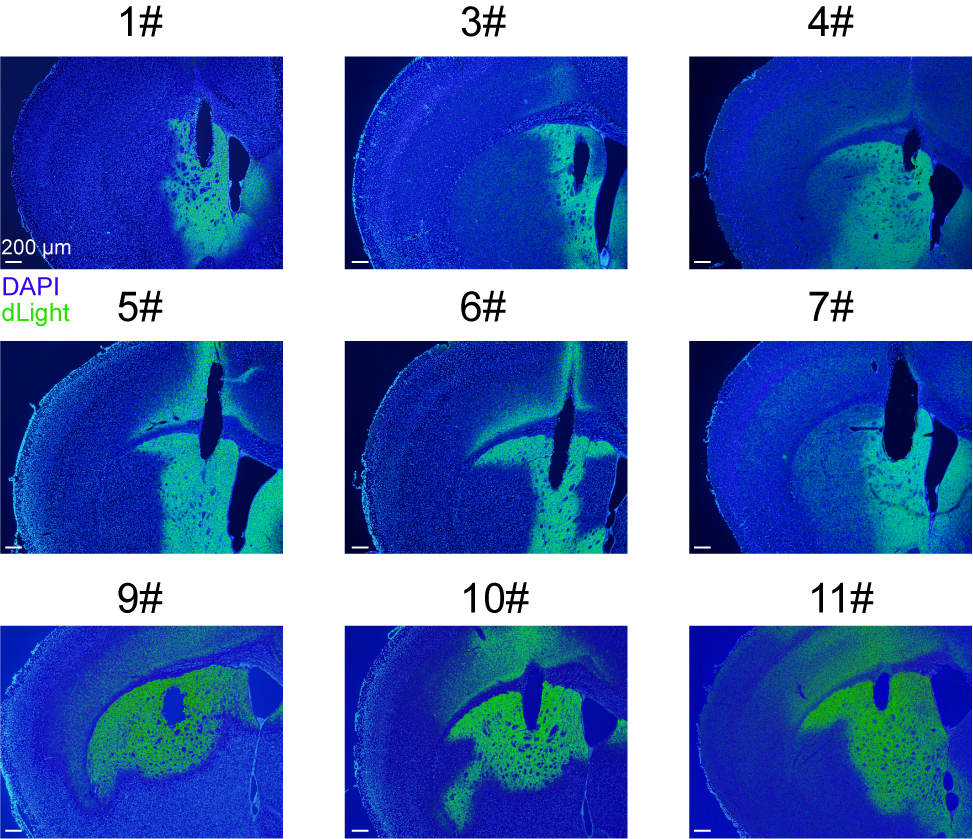

Supplement: FIGURE S1 — Expression of dLight1.1 in the dorsal striatum in all mice except 2# which is in Figure 1D. The blue is DAPI, the green is dLight1.1. The scale bar is 200 μm. [file Image_1.TIF]

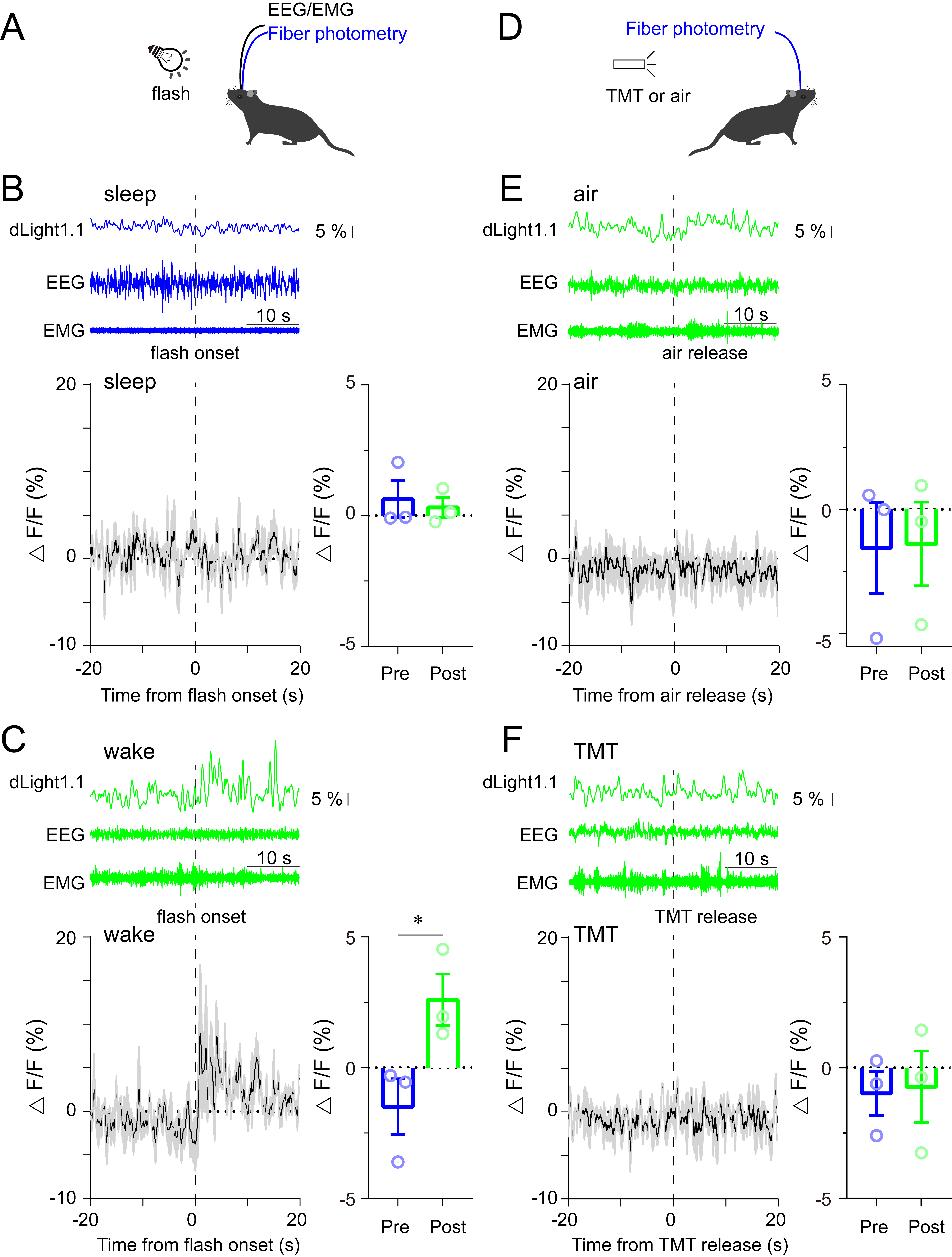

Supplement: FIGURE S2 — Striatal dLight1.1 fluorescence in response to light flash and predator odor. (A,D) Schematic showing light flash (A) or predator odor (D) was applied simultaneously with fiber photometry. (B) Up panel: Example traces of the fluorescence signal, EEG, and EMG before and after the onset of the light flash during sleeping. Down panel: (Left) The time course of the striatal dLight1.1 signal in response to light flash when mice were sleeping. (Right) Average fluorescence before and after onset of the light flash (t = 0.9181, P = 0.4555). (C) Up panel: Example traces of the fluorescence signal, EEG, and EMG before and after the onset of the light flash during awake. Down panel: (Left) The time course of the striatal dLight1.1 signal in response to light flash when mice were awake. (Right) Average fluorescence before and after onset of the light flash (t = 4.486, P = 0.0463). (E) Up panel: example traces of the fluorescence signal, EEG, and EMG before and after the onset of air release during awake. Down panel: (Left) The time course of the striatal dLight1.1 signal in response to air delivery. (Right) Average fluorescence before and after onset of the air delivery (t = 0.4803, P = 0.6784). (F) Up panel: example traces of the fluorescence signal, EEG, and EMG before and after the onset of TMT delivery during awake. Down panel: (Left) The time course of the striatal dLight1.1 signal in response to TMT delivery. (Right) Average fluorescence before and after onset of the TMT delivery (t = 0.2858, P = 0.8019). [file Image_2.TIF]

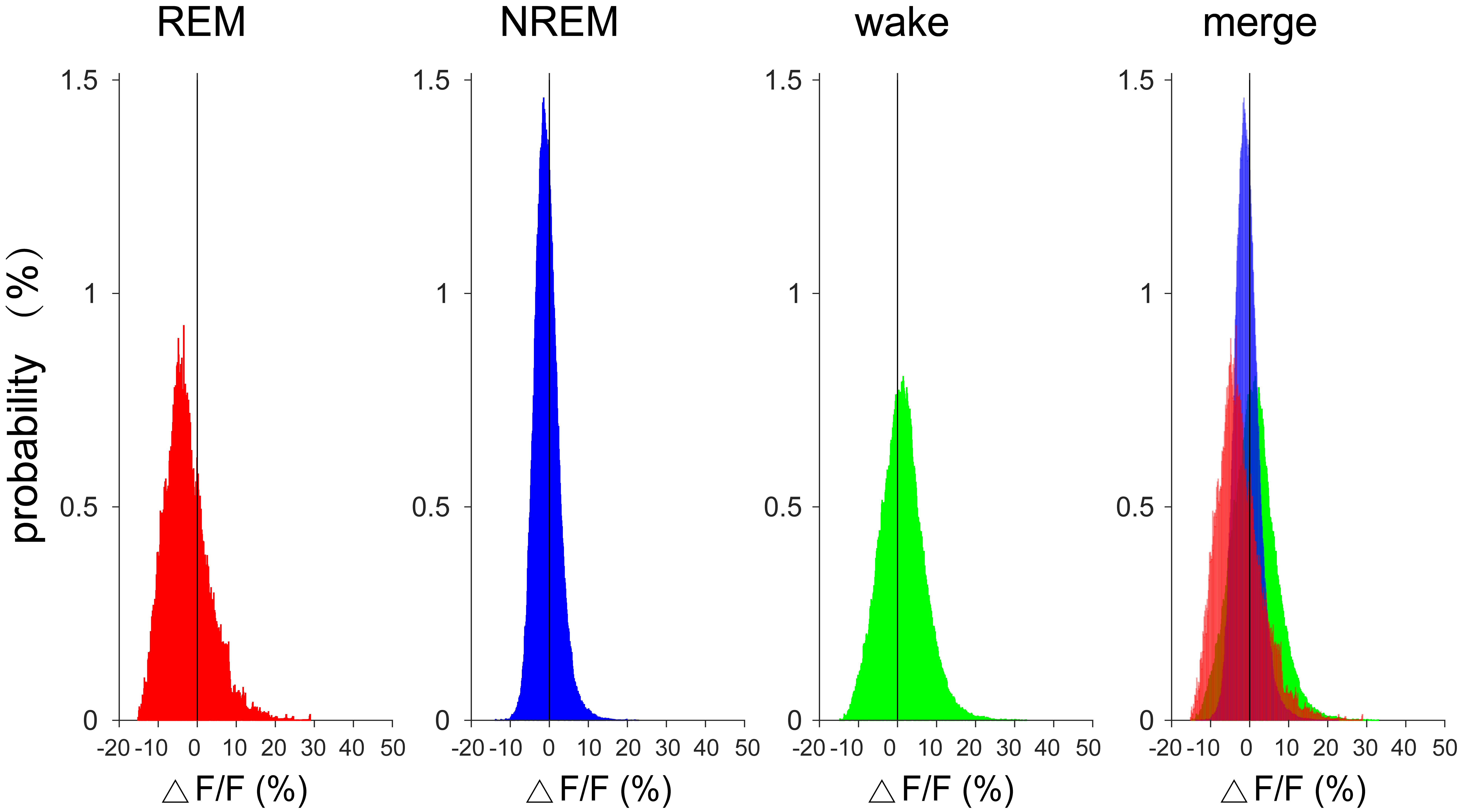

Supplement: FIGURE S3 — The distributions of dLight fluorescence values in each state. The red presents REM sleep, the blue for NREM sleep, and the green for wake. The fluorescence in REM sleep was more divergent, but more convergent in NREM sleep. There was 3.14% dLight fluorescence values in REM sleep higher than 0.1, 10.26% values higher than 0.05, 71.48% values lower than 0, and 7.51% values lower than -0.1, whereas there was 0.57% dLight fluorescence values in NREM sleep higher than 0.1, 4.42% values higher than 0.05, 62.18% values lower than 0, 0.04% values lower -0.1. Although it looks more higher in REM sleep because there a few higher values, the mean of dLight fluorescence was the lowest in REM sleep. [file Image_3.TIF]
